# Anemia and Heart Failure: A Narrative Review

**DOI:** 10.7759/cureus.27167

**Published:** 2022-07-23

**Authors:** Shiza W Siddiqui, Tejaswini Ashok, Nassar Patni, Mahejabeen Fatima, Aselah Lamis, Krishna Kishore Anne

**Affiliations:** 1 Research, Dubai Medical College, Dubai, ARE; 2 Internal Medicine, Jagadguru Sri Shivarathreeshwara (J.S.S) Medical College, Mysore, IND; 3 Internal Medicine, Deccan College of Medical Sciences, Hyderabad, IND; 4 Internal Medicine, National Pirogov Memorial Medical University, Vinnytsya, UKR

**Keywords:** anemia, iron deficiency anaemia (ida), anaemia of chronic disease (acd), epo, congestive heart faiulre, heart failure

## Abstract

Anemia in heart failure patients is a relatively common finding and has been linked with an increased risk of hospital admissions, morbidities, and significant mortality making its correction a significant factor in improving the quality of life and clinical outcomes in those suffering from it. This review article has discussed the multifactorial pathophysiology, including iron deficiency, longstanding inflammation, abnormal levels of human erythropoietin (Epo), and the abnormal activation of the renin-angiotensin-aldosterone system (RAAS) being the most significant. The diagnostic guidelines as well as research-based management modalities specifically with iron supplements and erythropoietin stimulating agents have also been discussed, although research done in this area has been limited and shown conflicting results.

## Introduction and background

Heart failure (HF) also termed congestive heart failure is the inability of the heart to pump blood to the body. According to the electronic data gathered by the Global Health Data Exchange (GHDx) registry, the global burden of HF stands at 64.34 million cases as of 2017, whereas the current global economic burden of HF can be measured at 346.17 billion US dollars, based on an American Heart Association (AHA) estimate of 5380 US dollars per heart failure case [1].

The World Health Organization classifies anemia as hemoglobin (Hb) levels <12 g/dL in women and <13 g/dL in men, however, the classification may differ when age, pregnancy status, altitude, and smoking status are considered [2]. The diagnostic criteria for anemia in heart failure patients are serum ferritin levels of less than 30 mcg/L in patients without kidney disease and less than 100 mcg/L in patients with chronic kidney disease or serum ferritin levels of 100-299 mcg/L with passing saturation of less than 20% in patients with chronic kidney disease [3].

The prevalence of anemia in heart failure patients ranges from 9% to 69.6% with an increased risk of hospital admissions and mortality in nearly 46.8% of patients in comparison to 29.5% in nonanemic patients [4]. The presence of other co-morbid medical conditions like chronic kidney disease (CKD) and advanced age as well as the severity of heart failure has also been associated with an increased prevalence of anemia [3].

The specific cause of anemia in heart failure patients is still unclear and has been thought to be multifactorial, with iron deficiency (IDA) and inflammation having the strongest evidence-based data [5].

The current treatment modalities in treating anemia in patients with heart failure include the use of erythropoietic agents - epoetin-α, epoetin-β, and darbepoetin-α and intravenous or oral iron supplementation [6]. The role of blood transfusion in patients with heart failure is controversial with different ‘’transfusion thresholds’’ in patients with cardiovascular diseases, debatable benefits in reducing mortality, and the presence of adverse side effects [7]. Blood transfusion can be viewed as an acute treatment for extreme anemia on an individual basis, but it does not appear to be a feasible therapeutic strategy for the long-term management of chronic anemia in CHF, based on the risk-benefit profile [7].

This review article aims to establish a relationship between anemia and heart failure as well as discuss screening techniques for early diagnosis and management in such cases.

## Review

Pathophysiology

The pathophysiology of anemia in heart failure patients is multi-factorial and has been linked to the presence of hematinic deficiencies, specifically iron deficiency (ID) anemia, chronic inflammation, or impaired erythropoietin levels. It can also be attributed to pseudo-anemia due to the activation of the renin-angiotensin-aldosterone system (RAAS).

1. Iron Deficiency Anemia (IDA)

Iron is one of the most important elements found in the human body. It is involved in erythropoiesis, transport, delivery, and utilization of oxygen, and is found in many enzymes responsible for crucial body functions [8]. Iron deficiency anemia in heart failure patients can be described as absolute or functional. Ferritin levels of < 100 μg/L or < 300 μg/L and low transferrin saturation (TSAT) of <20% have been used to diagnose heart failure patients with both absolute and functional ID [8].

Absolute iron deficiency is defined when total body iron levels are reduced and was identified in 15% of individuals with heart failure. [9,10]. This can be attributed to anorexia, cardiac cachexia, decreased iron absorption due to intestinal edema, hepcidin-induced downregulation of iron transporters such as ferroportin, gastrointestinal blood loss caused by aspirin, antiplatelet agents, or anticoagulants, as well as serious coexisting conditions like gastrointestinal or genitourinary malignancies [8,9].

Functional iron deficiency is described when iron levels are sufficient but not adequate to supply the target tissues due to maldistribution, Functional iron deficiency was found in 18% of heart failure patients. [8,10].

Iron deficiency anemia in heart failure patients is a common occurrence and is linked with severity, poorer prognosis, and outcomes as evidenced in an international, multicenter cohort study conducted by Klip et al. in 2013 with 1,506 participants with chronic heart failure. Out of these 1,506, 753 participants were found to be iron deficient and 426 anemics [9]. These patients were found to have a higher New York Heart Association (NYHA) class, higher prevalence of comorbidities, and increased levels of biomarkers as compared to patients with normal iron and hemoglobin levels [9].

Myocardial dysfunction has been linked to chronic ID due to changes in structure and function of the myocardium due to impaired oxygen metabolism, cellular activities, and immune mechanisms [11]. Impaired levels of reactive oxygen species (ROS) protective enzymes like catalase, glutathione peroxidase, and superoxide dismutase along with decreased mitochondrial oxygen consumption and reduced levels of aconitase and citrate synthase have been associated with reduced myocardial iron stores and impaired mitochondrial function in patients with heart failure [12]. Iron deficiency has also been linked to reduced exercise endurance, poor quality of life, and increased morbidity and mortality even without the presence of anemia [8].

2. Chronic Inflammation

Anemia of inflammation is the presence of normochromic normocytic anemia in the setting of an infection, inflammatory disease, or a malignant condition. A study conducted by Opasich et al. in 2005 established a relationship between anemia and congestive heart failure [13]. In this particular study, out of the 148 patients with stable heart failure (New York Heart Association class II and III) and hemoglobin concentrations of <13 g/dL (if males) or <12 g/dL (if females), 57% showed evidence of anemia of chronic disease, defined here as reduced concentrations of serum iron, transferrin, and total iron binding capacity; normal or raised ferritin; normal or slightly increased soluble transferrin receptor [13].

The pathophysiology behind this process is still largely unknown but is thought to be related to the increased levels of pro-inflammatory cytokines and hepcidin [14]. Endotoxin-induced immune activation due to bowel edema, myocardial production due to hemodynamic overload, and peripheral extra myocardial production due to tissue hypoperfusion and hypoxia have all been suggested as sources of cytokine production in heart failure [15]. Elevated levels of tumor necrosis factor alpha (TNF-alpha), interleukin (IL)-6, and IL-1 have been found in heart failure patients and are linked with a poorer prognosis and outcomes [16]. The surface of cardiomyocytes and fibroblasts are known to generate TNF-alpha and IL-6, whereas endothelial cells and interstitial macrophages are believed to be involved in the immunoreactivity of IL-1 [17-20].

**Figure 1:**
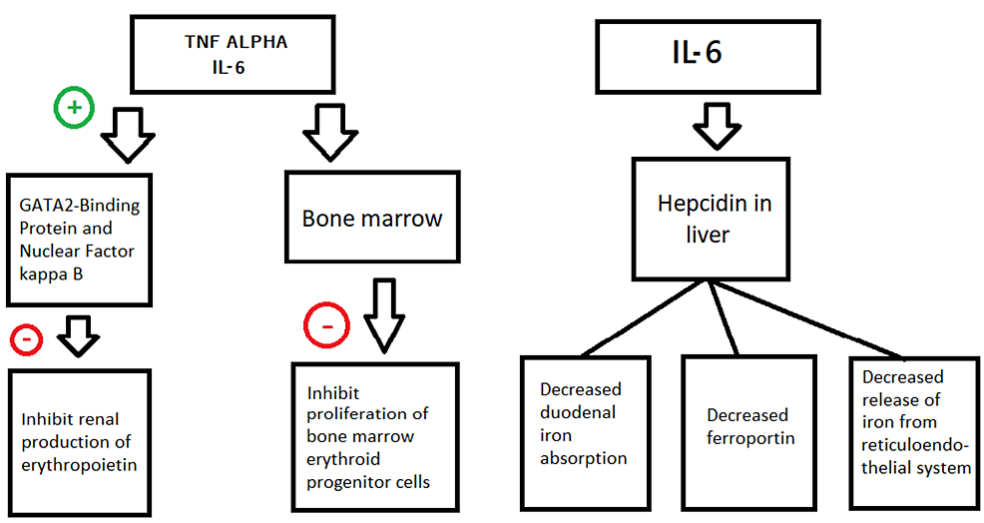
Effect of pro-inflammatory cytokines and hepcidin in the pathophysiology of anemia TNF - Tumor necrosis factor IL- interleukin Image Credits- Shiza Siddiqui

TNF-alpha and IL-6 have been shown to not only inhibit the production of erythropoietin in the kidneys via the activation of GATA 2 binding protein and nuclear factor-kB but are also involved in the suppression of erythroid progenitor cell proliferation in the bone marrow [21,22]. Additionally, IL-6 activates the synthesis of an acute phase protein, hepcidin in the liver which is involved in the downregulation of ferroportin [6]. Hepcidin also decreases the duodenal iron absorption and the release of iron from its stores in the reticuloendothelial system giving rise to functional and absolute iron deficiency anemia [5] (Figure 1).

3. Erythropoietin Levels

Human Erythropoietin (Epo) is a 169 amino acid long glycoprotein hormone with a molecular mass of 30.4 kDa synthesized primarily in the peritubular fibroblasts found in the renal cortex and the liver in a fetus [23]. Detectable levels have also been found in other organs such as the liver, spleen, bone marrow, lung, and brain [23]. The production of Epo is controlled by hypoxia-inducible transcription factors (HIF) and is mainly triggered in the presence of renal hypoxia and low concentrations of hemoglobin [23]. In heart failure patients, elevated levels of Epo inconsistent with the hemoglobin levels are found and are associated with an increased risk of morbidity and mortality [24].

The cause for the elevated levels is multifactorial. One of the proposed causes was the chronic inflammatory state associated with heart failure [25,26]. The release of pro-inflammatory cytokines has been linked with the impaired expression of Epo leading to an Epo resistance in the bone marrow ultimately resulting in elevated levels of endogenous Epo [27,28]. The production of Epo in response to angiotensin, despite the presence of angiotensin enzyme inhibitors, is another proposed cause [27]. The myocardium is one of the few non-renal tissues that can synthesize Epo in response to oxidative or metabolic stress or the presence of renal tissue impairment due to the presence of Epo receptors (EpoR) via the synthesis of HIF [5]. 

4. Renin-Angiotension-Aldosterone System (RAAS)

**Figure 2:**
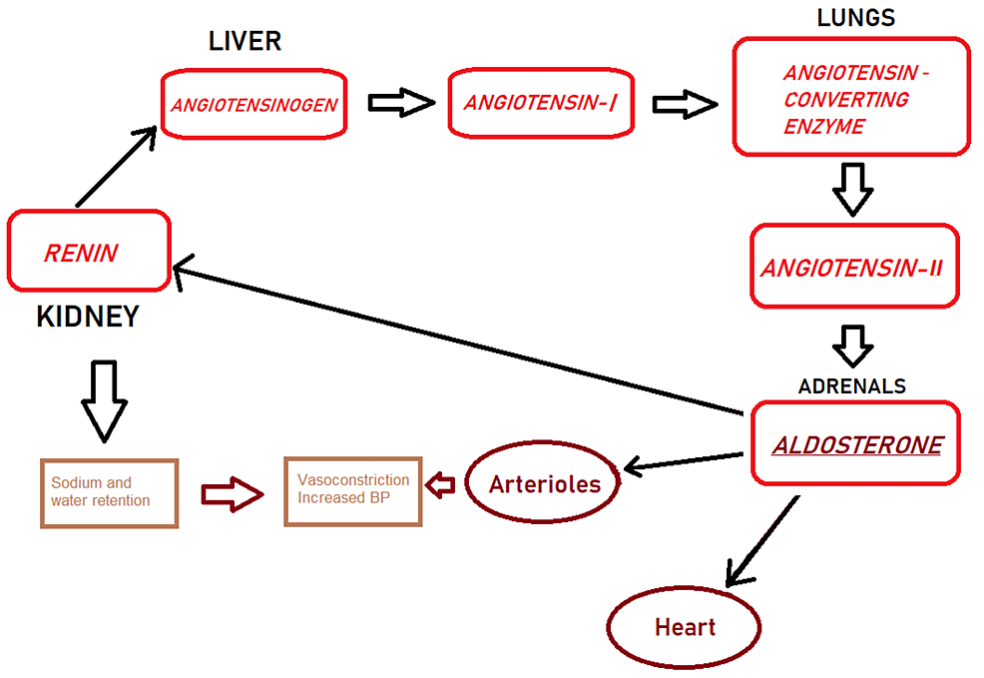
Renin- angiotensin-aldosterone system BP- Blood pressure Image Credits- Shiza Siddiqui

The RAAS is a hormonal system that is activated in response to renal hypoxia leading to the cleavage of angiotensinogen to form angiotensin I in the liver, and then angiotensin II by the angiotensin-converting enzyme (ACE) predominantly in the lungs [28]. Angiotensin II then further activates aldosterone which causes increased sodium uptake from the renal tubules and increases the extracellular volume and blood pressure [28] (Figure 2).

The renin-angiotensin-aldosterone system (RAAS) is an important mediator in the pathophysiology of heart failure [29,30]. The increased activity of RAAS in heart failure, although initially improves cardiac output, however, over time is associated with various adverse effects such as cardiac remodeling and sympathetic nervous system activation and progressively worsens the condition [29,30]. The use of angiotensin-converting enzyme inhibitors (ACEIs) and angiotensin receptor blockers (ARBs) is a major tool in the management of heart failure, but it may hamper the hemoglobin levels due to the effect of angiotensin II on erythropoietin synthesis and the erythroid progenitor cell production in the bone marrow [8]. Moreover, ACEIs are associated with a modest decrease in hemoglobin levels due to the inhibition of the breakdown of N-acetyl-seryl-aspartyl-lysyl-proline (Ac-SDKP), a hematopoiesis inhibitor [31]. 

5. Other Causes

Anemia in heart failure patients may be due to haemodilution brought upon as a consequence of sodium and water retention due to the activation of the RAAS pathway [32]. The use of medications such as beta-blockers (particularly carvedilol) and digoxin have shown to be associated with reduced hemoglobin levels [5,33].

The clinical dilemma of anemia in heart failure patients

Anemia in heart failure patients has been linked with an increased risk of hospital admissions and mortality as supported by studies conducted by Schou et al. (2007) [34] and Komajda et al. (2006) [35] where similar criteria were used to define anemia (according to WHO criteria) and LVEF <45% and LVEF ≤35%, respectively were taken into account (Table 1). Both studies came to a similar conclusion that anemia in heart failure patients was associated with a markedly increased risk of mortality [34,35].

A study conducted by Kosiborod et al. (2005) included 50,405 participants previously diagnosed with heart failure evidenced either by history or radiographic findings. An increased risk of one-year mortality rate and morbidities due to heart failure in patients with lower hematocrit was found when anemia was defined as < 40% for men and < 37% for women [36].

Another group of studies substantiated by Anand et al. (2004) as part of the Randomized Etanercept North American Strategy to Study Antagonism of Cytokines (RENAISSANCE) trial, found the presence of anemia (Hb ≤12.0 g/dL) in 12% of the 69 participants [37]. The participants all belonged to NYHA classes II to IV and had LVEF < 30% and were 18-55 years old [38]. The study found that the risk of mortality was 15.8 percent lower for every 1 g/dL greater baseline Hb, and the risk of mortality or hospitalization for heart failure was 14.2 percent lower [37]. Furthermore, it was discovered that anemic participants (Hb 12.0 g/dL) had a much higher mortality rate than nonanemic participants (28 percent against 16 percent, respectively) [37].

Ezekowitz et al. (2003) [38], in another study conducted among 12,065 patients diagnosed with heart failure, according to the International Classification of Diseases (ICD)-9 code 428.x, found the presence of anemia (ICD-9 codes 280-289) in 17% percent of the participants [39]. Among these participants, Iron deficiency accounted for 21%, other deficiencies accounted for 8%, miscellaneous other identified causes accounted for 13%, and anemia of chronic disease accounted for 58% [38]. The study found the presence of higher one-year and five-year mortality rates among those with anemia (38% and 59% respectively) as compared to those without anemia (27% and 50% respectively) [38]. 

A total of 30% out of 1,061 patients were found to have anemia (as Hb <13 g/dl in men and Hb <12 g/dl in women) in a study conducted by Horwich et al. (2002) [40]. All the participants had heart failure defined by the presence of NYHA class III and IV and an LVEF <40%. Poor outcome in the form of mortality was accounted for in 212 participants by the end of one year and 360 at the end of five years [40].

Anand et al. [37] and Horwich et al. [40] evidenced the association of anemia in patients with a higher form of heart failure (higher NYHA classification, lower LVEF,) with poorer outcomes and higher mortality rates based on a similar diagnostic criterion for diagnosing anemia [37,40].

Even though both studies conducted by Ezekowitz et al. and Horwich et al. used different classification criteria for the diagnosis of heart failure and anemia, both deduce higher one-year and five-year mortality rates among anemic heart failure patients as compared to their non-anemic counterparts thus establishing that anemia in heart failure patients is associated with poorer outcomes [38-40].

Anemia has also been proposed as a marker for mortality in heart failure patients as evidenced by cohort studies conducted by Kosiborod et al. (2005) [36] and Ezekowitz et al. (2003) [38]. While Kosiborod et al. defined anemia according to hematocrit and Ezekowitz et al. in accordance with ICD-9 codes, both the studies came to a similar conclusion that anemia is an important marker for the prognosis, severity, and mortality in heart failure patients [36,38].

Population data table

Table 1 below compares various studies /researches/ trials conducted depicting the relationship between anemia and heart failure amongst various populations.

**Table 1:** Population data table LVEF- Left ventricular ejection fraction; Hb- Hemoglobin; HF- Heart failure; WHO- World Health Organization; COMET- Comparing an Operation to Monitoring, With or Without Endocrine Therapy; NYHA- New York Heart Association; ICD- International classification of diseases Klip et al. (2013) [9]; Schou et al. (2007) [34]; Komajda et al. (2006); [35] Kosiborod et al. (2005) [36]; Anand et al. (2004) [37]; Ezekowitz et al. (2003) [38]; Horwich et al. (2002) [40]

References	Study design	Population and setting	Cases of HF	Prevalence of anemia	HF classification	Criteria for diagnosis of anemia	Conclusion
Klip et al. (2013)	Prospective observational, multicenter cohort study	Patients from 5 cohorts from Poland, Spain and The Netherlands	1,506	426	Reduced or preserved LVEF	Hb <13 g/dL for men and <12 g/dL for women (WHO criteria) [2]	Iron deficiency is a common finding in HF patients and is associated with the severity of the disease.
Schou et al. (2007)	Prospective, observational, single-center cohort study	Outpatients with Systolic Heart Failure in Frederiksberg University Hospital, Denmark	345	27%	Systolic HF (LVEF <45%)	WHO criteria	Anemia is associated with an increased risk of mortality in HF patients.
Komajda et al. (2006)	Secondary analysis of clinical trial (COMET)	Participants of the COMET trial	3029	482	NYHA class II to IV and LVEF ≤35%	WHO criteria	Anemia is associated with increased hospital admissions and mortality in HF patients.
Kosiborod et al. (2005)	Retrospective observational, multicenter cohort study	Age of 65 years and older from NHC Project sample	50,405	Men -61.2% Women- 52.1%	History of documented HF. Radiographic evidence of HF.	Hematocrit of < 40% for men and < 37% for women.	Anemia is associated with heart failure and may be a marker of increased mortality in HF patients.
Anand et al. (2004)	Multicenter, double-blind, placebo-controlled RENAISSANCE trial	Age of 18 to 55 years in North America	912	12%	NYHA class II to IV	Hb≤12.0g/dL	Anemia is a common finding in patients with HF and is associated with poorer prognosis and greater mortality rates.
Ezekowitz et al. (2003)	Population-based cohort study	Median age of 78 in Alberta, Canada	12,065	17%	ICD-9 Code 428.x [39]	ICD-9 codes 280-289 [39]	Anemia is a common finding in patients with heart failure and is associated with poorer prognosis and mortality.
Horwich et al. (2002)	Prospective, observational, single-center cohort study	Patients referred to a single University Medical Center in University of California, Los Angeles, USA	1,061	30%	NYHA class III or IV and LVEF <40%	WHO criteria	Anemia is associated with poorer prognosis and greater mortality rates in patients of HF.

Diagnostic guidelines of anemia in heart failure

As per the current 2016 European Society of Cardiology (ESC) heart failure guidelines, class of recommendation I, and level of evidence C, the diagnostic investigations for newly diagnosed patients of heart failure should include the iron status, particularly the ferritin and transferrin saturation (TSAT) levels. The guidelines also recommend, although partially, the evaluation of iron levels in already diagnosed patients of HF, especially if they are symptomatic. Additionally, the guidelines also recommend the inclusion of iron status as part of the routine follow-up and post-discharge [41,42].

Serum ferritin and TSAT are markers for the quality and availability of stored iron and are valuable indicators of iron status [42]. Serum ferritin levels < 100 µg/L, or 100-299 µg/L with TSAT < 20% are indicative of iron deficiency according to the 2016 ESC HF guidelines [41]. The levels of mean corpuscular volume (MCV) and the mean corpuscular (MCH) concentration are not recommended as they are unreliable markers in the assessment of iron deficiency in heart failure patients [43]. Additionally, serum iron alone is also an unreliable marker due to its variable nature and is recommended to be used as a screening tool with serum ferritin and TSAT [44].

Management of anemia in heart failure: a dilemma

Anemia in heart failure is associated with poor outcomes and mortality, as discussed earlier, making its correction a reasonable part in the management of the disease and improving its outcomes. A few modalities are available, although researches done in this area are limited and have shown conflicting results, these include the following

 1. RBC Transfusion

The role of transfusion in the correction of hemoglobin levels in heart failure patients has been shown to only offer a temporary benefit with an additional risk of multiple adverse effects, making its usage limited and unsuitable for long-term management [45].

 2. Iron Supplementation

Since iron deficiency is a major cause of anemia in heart failure patients, management with iron supplements - oral (PO) and intravenous (IV) is worth looking into. Unfortunately, not many studies have been done in regards to the usage and benefits of oral iron therapy, mostly due to the reduced gastrointestinal absorption due to the high hepcidin levels seen in heart failure and its known gastrointestinal complications impacting its compliance [5].

One of the most recent trials conducted to assess the efficacy of oral iron supplements in patients with heart failure suffering from iron deficiency anemia is the IRONOUT-HF Randomized Clinical Trial [46]. The trial was a Phase 2, double-blind, placebo-controlled randomized clinical trial with 225 participants that defined heart failure as LVEF< 40% and iron deficiency as serum ferritin level between 15-100 ng/ml or serum ferritin 101-299 ng/ml with transferrin saturation (TSAT) <20% [46]. It included change in peak oxygen uptake (VO2), from baseline to 16 weeks, changes in 6-minute walk distance; plasma NT-pro BNP (N-terminal-pro hormone B-type natriuretic peptide) levels; and health status as assessed by Kansas City Cardiomyopathy Questionnaire (KCCQ) as its endpoints [46]. The trial established the lack of improvement in exercise capacity after the use of a high-dose oral iron regimen and did not support the use of it in heart failure patients [46].

The use of intravenous (IV) iron is preferred over the use of oral iron due to its superiority as established by the IRON-HF study, a multicenter, investigator-initiated, randomized, double-blind, placebo-controlled trial [47]. The trial compared the efficacy of oral iron versus IV iron in 23 patients who received either Iron Sucrose IV, ferrous sulfate PO, or placebo, in a randomized manner [47].

A double-blind, randomized, placebo-controlled study to evaluate changes in levels of NT-pro-brain natriuretic peptide (NT-proBNP) and C-reactive protein (CRP) levels in patients receiving IV iron therapy without recombinant human erythropoietin (rhEPO)[48]. All 40 participants with anemia (hemoglobin (Hb) <12.5 g/dl, transferrin saturation <20%, ferritin <100 ng/ml) in a setting of chronic heart failure (LVEF < or =35%) or chronic renal failure (creatinine clearance <90 ml/min) were either given IV iron therapy or a placebo, at random, for five weeks after which they were evaluated on the based on Minnesota Living with Heart Failure Questionnaire (MLHFQ) and 6-min walk (6MW) test [48]. The study concluded that the use of IV iron therapy in absence of rhEPO resulted in the betterment of LVEF, NYHA class, exercise capacity, and general quality of life [48].

One of the most recent trials conducted to understand the benefit of IV iron therapy in patients with heart failure is the AFFIRM-AHF study [49]. This multicenter, randomized, double-blind, placebo-controlled trial investigated the benefits of IV ferric carboxymaltose (FCM) in patients hospitalized due to acute heart failure [49]. The trial included 1,108 patients with LVEF< 50% after receiving at least 40 mg IV furosemide (or similar) and having indications of iron deficiency (serum ferritin <100 ng/mL or ferritin 100-299 ng/mL with transferrin saturation <20%) after initial clinical stabilization [49]. The trial concluded that the use of IV iron reduces hospitalizations and improves symptoms and functional capacity with no change in mortality in patients with iron deficiency in the setting of acute decompensated heart failure [49].

Hence, IV iron might be considered as a treatment modality for anemia in heart failure patients to improve the quality of life and functional capacity, with no change in mortality as recommended by the American College of Cardiology/American Heart Association in 2017 [50].

 *3. Erythropoiesis-Stimulating Agents*

The use of erythropoiesis-stimulating agents (ESA), specifically recombinant human erythropoietin- epoetin-alfa and epoetin-beta and the analogue darbepoetin alfa, has been evidenced in improving anemia in chronic kidney disease and malignancies prompting exploration of their potential effects on the treatment of anemia due to heart failure [51].

A meta-analysis comprising of 13 randomized clinical trials to assess the impact of ESA on the mortality and hospitalization in heart failure patients with anemia concluded that although ESA improved the NYHA class and quality of life, it had an insignificant effect on the mortality and hospitalization rate [52]. Not to mention, the overall risk for thromboembolic events was observed [52].

Another similar meta-analysis that included 11 studies comparing the ESA therapy with placebo found improved levels of hemoglobin, increased LVEF, a decrease in B-type natriuretic protein, and an improved NYHA class in the group receiving ESA [53]. The meta-analysis also noted a decrease in the rate of hospitalization but no change in the rate of mortality [53]. The analysis concluded that the use of ESA therapy improved the symptomatology but did not have any change in the clinical outcomes of anemic heart failure patients [53].

The RED-HF (Reduction of Events with Darbepoetin alfa in Heart Failure) trial, conducted in 2013, was a randomized, double-blind trial evaluating the effects of darbepoetin alfa in patients with systolic heart failure and anemia in regard to the clinical outcomes [54]. The trial comprised of 2,278 patients, with NYHA class II, III or IV; an LVEF < or = 40% and a hemoglobin of 9-12 g/dL, that were randomly given either darbepoetin alfa or placebo in a 1:1 ratio [54]. It was noted that the treatment of anemia with darbepoetin alfa improved the Overall Summary and Symptom Frequency Scores on the Kansas City Cardiomyopathy Questionnaire (KCCQ) however there was no reduction in the rate of mortality or hospitalization [54]. Furthermore, a significant increase in the frequency of thromboembolic events was noted in the darbepoetin alfa group [54]. The trial advocated against the use of darbepoetin alfa in patients with systolic heart failure and anemia [54].

One of the largest trials evaluating the effect of treating anemia in heart failure patients with darbepoetin alfa is the Study of Anemia in Heart Failure Trial (STAMINA-HeFT), a multicenter, randomized, double-blind, placebo-controlled trial published in 2008 [55]. Similar to the RED-HF trial, this trial also compared a placebo group to the group receiving darbepoetin alfa based on clinical benefits and outcomes [54,55]. No significant changes in exercise duration, NYHA class, or quality of life were noted upon receiving darbepoetin alfa contrary to that observed in the RED-HF trial [54,55]. However, a lower risk of morbidity and mortality was noted as opposed to the RED-HF trial [54,55].

As per the guidelines set by the American College of Cardiology/American Heart Association in 2017, ESA should not be used in patients with heart failure and anemia to improve morbidity and mortality [50].

## Conclusions

Anemia in heart failure patients is a significant finding and is related to poor outcomes and increased morbidity and mortality, as evidenced by the article. The aim of this article is to establish a link between the pathophysiology and the implications of anemia in patients suffering from heart failure along with a brief outlook on the treatment modalities for such patients.

This article not only establishes the association of iron deficiency, chronic inflammation, erythropoietin levels, and the renin-angiotensin-aldosterone system with the development of anemia in heart failure patients but also sheds a light on the latest developments in the management, with the help of multiple evidence-based researches, of such patients. Intravenous iron supplementation was found to improve the quality of life and functional capacity, with no mortality benefit whereas studies revealed that the use of erythropoiesis-stimulating agents (darbepoetin alfa) in heart failure patients had little to no benefit in improving morbidity and mortality with the latest guidelines recommending against its use in these patients.

We believe that this review article can provide valuable information regarding this complex topic. Furthermore, this article establishes the importance of screening and approach in the management of such patients keeping the NYHA class, hemoglobin levels and the clinical picture in mind. This review compiles the different diagnostic guidelines as per the latest recommendations by the European Society of Cardiology (ESC), 2016 and compiles the different researches in this field. Lastly, we believe further research in this complex yet important and clinically significant topic is necessary to provide a better understanding of the association between anemia and heart failure and a more systematic approach towards its diagnosis and management.
